# Preliminary Investigation of Disorders of Gut–Brain Interaction and Their Associations With Psychological Flexibility and Daily Functioning in People With Fibromyalgia

**DOI:** 10.1002/ejp.70378

**Published:** 2026-09-03

**Authors:** Lin Yu, Kitty Kioskli, Alice Sibelli, Lance M. McCracken

**Affiliations:** ^1^ Department of Psychology Middlesex University London UK; ^2^ Trustilio, B.V. Amsterdam the Netherlands; ^3^ Sibelli Consultancy London UK; ^4^ Department of Psychology Uppsala University Uppsala Sweden; ^5^ Centre for Biomedical Research in Epidemiology and Public Health (CIBERESP) Madrid Spain

**Keywords:** comorbidity, disorders of gut–brain interaction, fibromyalgia, functioning, psychological flexibility

## Abstract

**Background:**

A substantial proportion of people with fibromyalgia also experience comorbid disorders of gut–brain interaction (DGBIs), which can lead to considerable personal and societal burdens. However, evidence regarding the implications of this comorbidity remains limited. The aim of this study was to investigate the occurrence of DGBIs in fibromyalgia, potential consequences for people when this happens, and a process called psychological flexibility (PF) that might help.

**Methods:**

395 adults with fibromyalgia completed an online survey and were included in the analyses. Analyses included group comparisons, correlations, and mediation models.

**Results:**

79.2% of the participants reported having at least one DGBI. Participants with multiple DGBIs reported significantly worse daily functioning compared with those without any DGBI. More severe gastrointestinal (GI) symptoms were correlated with more severe depression and worse daily functioning (*r* = 0.41 to 0.56, *p* < 0.001). PF statistically mitigated the relationship between GI symptom severity and depression and daily functioning (*b* = 0.05–0.13).

**Conclusions:**

While people with comorbid fibromyalgia and DGBIs report worse functioning, PF appears to modestly buffer the relationship between DGBI symptom severity and mood and daily functioning. Therefore, treatments that target PF, such as Acceptance and Commitment Therapy (ACT), may help address the challenges faced by this population. Longitudinal studies with experimental designs are needed to further explore the mediating role of PF and the utility of related treatments for this population.

**Significance Statement:**

This study investigates disorders of gut–brain interaction (DGBIs) in relation to psychological flexibility and functioning in people with fibromyalgia, and the prevalence of DGBIs in fibromyalgia in a large community sample. The findings show a high rate of co‐occurring DGBIs and fibromyalgia. While the size of the effect was limited, psychological flexibility appears to act as a buffer in the relationship between symptom severity and functioning, suggesting that interventions targeting psychological flexibility may provide benefits for co‐occurring conditions.

## 1 Introduction

Fibromyalgia is a complex, long‐term condition affecting up to 4.43% of the general population (Heidari et al. 2017). People with fibromyalgia experience impaired daily functioning, reduced work productivity, and lower quality of life (D'Onghia et al. 2022). A substantial proportion of people with fibromyalgia also experience comorbid disorders of gut–brain interaction (DGBIs), previously known as functional gastrointestinal disorders, with prevalence estimates as high as around 90% (Erdrich et al. 2020; Erdrich and Harnett 2025).

DGBIs are chronic gastrointestinal (GI) conditions not explained by structural abnormalities, and several mechanisms have been proposed to elucidate the underlying dysregulation of the brain–gut axis including abnormal gut motility, visceral hypersensitivity, altered mucosal and immune function, gut microbiota imbalance, and disrupted central nervous system processing (Keefer et al. 2022). These conditions present unique challenges, such as worries about incontinence, sudden urgency, or audible bowel sounds in public, which can result in social withdrawal and isolation (Taft and Keefer 2016). Having multiple DGBIs is associated with more severe fibromyalgia, pain, and sleep disturbances (Erdrich and Harnett 2025). Having multiple DGBIs is also associated with more severe symptoms, more psychological comorbidities, increased healthcare utilisation, and lower quality of life (Barbara et al. 2025; Sperber et al. 2021).

Despite the high prevalence and impact of comorbid fibromyalgia plus DGBIs, evidence regarding the associations between DGBIs, fibromyalgia and various aspects of functioning, including physical, emotional and overall daily functioning, remains narrowly focused on Irritable Bowel Syndrome (IBS) (Erdrich and Harnett 2025), the most common DGBI in people with fibromyalgia. Evidence regarding protective factors is limited.

A psychological process called psychological flexibility (PF) may play a role in the impact of the co‐occurring conditions. The PF model includes six interrelated processes, namely acceptance, cognitive defusion, present awareness, self‐as‐context, values and committed action. Some PF processes, including acceptance and self‐as‐context, have been found to be associated with functioning in people with fibromyalgia (Yu et al. 2017) and mixed GI conditions (Yu et al. 2021). Additionally, several studies have shown the improvements in PF processes following Acceptance and Committed Therapy (ACT; Hayes et al. 2011) for fibromyalgia (Gendreau et al. 2024; Luciano et al. 2014; Simister et al. 2018; Wicksell et al. 2013) and ACT for IBS (Ferreira et al. 2018). However, PF has not been examined in people with co‐occurring fibromyalgia and DGBIs.

The aim of this study was to investigate the occurrence of DGBIs in fibromyalgia, potential consequences for people when this happens, and psychological processes that might help. This included three objectives: (1) To preliminarily examine the rate of individuals with DGBIs in individuals with fibromyalgia; (2) To examine the associations between condition‐related burden and PF and indicators of mood and daily functioning, including the functional impact of fibromyalgia, work and social functioning, and depression; (3) To explore whether PF might provide a buffer in the relationship between GI symptom severity and mood and daily functioning.

## 2 Methods

### 2.1 Design and Procedures

This study included a cross‐sectional online survey of people with fibromyalgia delivered via the online survey platform Survey Monkey. Ethical approval was obtained for this study from Middlesex University Psychology Research Ethics Committee, on 3rd March 2025 (Application number: 29945). Informed consent was obtained from all participants.

The public was actively involved in the recruitment process. The study was discussed with relevant online support groups and charities that support people with fibromyalgia, regarding the relevance and benefit of the study and potential involvement in the recruitment of the study. Advertisement for the study with the link for the online survey was then widely distributed via online social media platforms, including the online support groups (e.g., Fibromyalgia Research UK Facebook group: https://www.facebook.com/FRUKgroup/) and charities (e.g., Fibromyalgia Action UK) that agreed to be involved in the recruitment of the study.

Potential participants clicked on the link for the online survey to view detailed information about the study and answered questions regarding eligibility. Individuals who were at least 18 years old with self‐reported fibromyalgia were deemed eligible. Eligible potential participants provided informed consent and continued to participate if they wished to. Participants were shown a debriefing sheet at the end of the survey, where relevant supporting resources were provided.

### 2.2 Participants

437 individuals were screened for eligibility. Six individuals were excluded as they did not have fibromyalgia. Out of the 431 eligible individuals, 424 provided consents. Among the 424 participants, 29 participants did not provide any data on any of the measures of mood and daily functioning and were excluded from analyses. 395 participants were included the in analyses. Table 1 shows the characteristics of the participants.

**TABLE 1:** Characteristics of participants.

Variable	Subcategory	Frequency (%) or Mean (SD)
Gender	Women	383 (97%)
	Men	11 (2.8%)
	Other	1 (0.3%)
Age		50.51 (10.8%)
Ethnicity	White	381 (96.5%)
	Mixed	6 (1.5%)
	Asian	4 (1.0%)
	Black	3 (0.8%)
	Other	1 (0.3%)
Education (Years)		15.08 (5.44)
Working status	Unemployed due to pain	149 (37.7%)
	Working/studying full‐time	87 (22%)
	Working/studying part‐time due to pain	74 (18.7%)
	Retired	46 (11.6%)
	Unpaid work (e.g., volunteer, carer and homemaker)	18 (4.6%)
	Working/studying part‐time for other reasons	12 (3%)
	Unemployed for other reasons	9 (2.3%)
Fibromyalgia diagnosis	Diagnosed by a clinician	389 (98.5%)
Pain duration (Years)		16.64 (11.77)
Generalised pain		356 (90.1%)
Primary pain site	Lower back, lumbar spine, sacrum and coccyx (tailbone)	140 (35.4%)
	Lower limbs	96 (24.3%)
	Upper shoulder or upper limbs	75 (19%)
	Neck region	33 (8.4%)
	Abdominal (stomach) region	19 (4.8%)
	Pelvic region	12 (3%)
	Head, face or mouth	9 (2.3%)
	Chest region	8 (2%)
Mood and daily Functioning	Functional impact of Fibromyalgia (FIQ‐R)	69.53 (17.54)
	Work and social functioning (WSAS)	30.88 (7.44)
	Depression (PHQ‐9)	15.90 (5.94)
Symptom severity	Gastrointestinal symptom severity (GSRS)	12.38 (3.56)
	Fibromyalgia symptom severity (symptoms domain of FIQ‐R)	69.06 (16)
Psychological flexibility (Psy‐Flex)		292.07 (131.59)

Abbreviations: GSRS, Gastrointestinal Symptoms Rating Scale; FIQ‐R, Fibromyalgia Impact Questionnaire‐Revised; PHQ‐9, Patient Health Questionnaire‐9; WASA, Work and Social Adjustment Scale.

### 2.3 Measures

#### 2.3.1 Background Information

Participants were asked about their demographic information including age, gender, ethnicity, working status, education. Participants were also asked about pain including pain intensity, pain duration, and pain location. Additionally, participants were asked to indicate any diagnoses of DGBIs based on the Rome Foundation Disorders of Gut–Brain Interaction Definitions they had received.

#### 2.3.2 Pain Scale

Pain intensity was assessed through one question using a 0 to 10 numerical rating, where 0 indicates ‘no pain’ and 10 indicates ‘pain’ as bad as you can imagine, in relation to their average pain in the past two weeks (Jensen et al. 1999; Von Korff et al. 1992).

#### 2.3.3 Fibromyalgia Impact Questionnaire‐Revised (FIQ‐R)

The Fibromyalgia Impact Questionnaire is a commonly used measure to assess the functional impact of fibromyalgia (Burckhardt et al. 1991). The revised version of this measure was used in this study (FIQ‐R) (Bennett et al. 2009). It includes 21 questions clustered in three domains including function, overall impact, and symptoms. The function domain includes nine items assessing the extent to which fibromyalgia interferes with everyday tasks. The overall impact domain includes two items assessing the overall impact of fibromyalgia (difficulty achieving goals, feeling overwhelmed by symptoms). The symptoms domain includes 10 items assessing fibromyalgia symptom severity. All item responses are based on an 11‐point numeric rating scale of 0 to 10, with 10 indicating ‘worst’, and all questions are framed in the context of the past 7 days. A summary score is calculated for each domain, and a total score is calculated from the summary scores. The reliability of the scale in this study was good to excellent for each subscale, *α* = 0.94, 0.85 and 0.86, and excellent for the total scale, Cronbach's *α* = 0.95.

#### 2.3.4 Gastrointestinal Symptoms Rating Scale (GSRS)

The GSRS is a reliable and valid disease‐specific measure of GI symptom severity (Kulich et al. 2008). Fifteen items are combined into five symptom clusters including Reflux, Abdominal pain, Indigestion, Diarrhoea, and Constipation. Symptoms during the past week are assessed on a seven‐point scale from 1 to 7, where 1 indicates ‘no discomfort at all’, and 7 indicates ‘very severe discomfort’. An average score was calculated from the summary score for each of the five symptom clusters to reflect overall GI symptom severity. The reliability of the scale in this study was good, Cronbach's *α* = 0.86.

#### 2.3.5 Work and Social Adjustment Scale (WSAS)

The WSAS is a reliable and valid five‐item measure of social and occupational functioning, which includes functioning in work, home management, private and social leisure activities, and close relationships (Mundt et al. 2002). Each item is rated on a scale from 0 to 8, 0 indicating ‘no impairment’ and 8 indicating ‘very severe impairment’. Scores for all five items were summed to produce a total score, with higher scores indicating greater functional impairment. A total score below 10 is associated with sub‐clinical populations, 10–20 associated with significant functional impairment, and 20 and above associated with moderately severe or worse functional impairment. The reliability of the scale in this study was good, Cronbach's *α* = 0.86.

#### 2.3.6 Patient Health Questionnaire‐9 (PHQ‐9)

The PHQ‐9 is a ten‐item self‐report measure of depression severity (Kroenke et al. 2001). The first nine items assess symptoms of depression and are rated on a scale from 0 to 4, where 0 indicates ‘not at all’ and 4 indicates ‘nearly every day’. The last item assesses the impact of depression and is rated on a scale from 0 ‘not difficult at all’ to 4 ‘extremely difficult’. The total score of the first nine items was used in the study to reflect the severity of depression, with higher score reflecting more severe depression. The standard clinical interpretive thresholds for the PHQ‐9 are: 0–4 = none to minimal depression, 5–9 = mild depression, 10–14 = moderate depression, 15–19 = moderately severe depression, and 20–27 = severe depression. In the current sample, the PHQ‐9 demonstrated good internal consistency, Cronbach's *α* = 0.86.

#### 2.3.7 Psy‐Flex

The Psy‐Flex is a six‐item self‐report questionnaire assessing all six processes of psychological flexibility, including acceptance, cognitive defusion, present moment awareness, stable self‐awareness, values, and committed action (Gloster et al. 2021). The Psy‐flex has recently been validated in a large chronic pain sample (Chia 2024), demonstrating reliability (Cronbach's *α* = 0.86), convergent validity, evidenced by significant negative correlations with a measure of psychological inflexibility (r = −0.61 to −0.49), and predictive validity evidenced by significant correlations with pain (r = −0.11, *p* < 0.05), pain interference (r = −0.34, *p* < 0.01), pain catastrophising (r = −0.45, *p* < 0.01) and depression (r = −0.57, *p* < 0.01). Participants were asked to rate each of the items on the scale from 0 ‘strongly disagree’ to 100 ‘strongly agree’, based on their experience in the past week. One example item is ‘Even if I am somewhere else with my thoughts, I can focus on what's going on in important moments’. Scores for all items were summed to produce a total score, with higher scores indicating greater PF. In the current sample, the Psy‐Flex demonstrated good internal consistency, Cronbach's *α* = 0.85.

### 2.4 Statistical Analysis

*T*‐test was conducted to examine whether participants with at least one DGBI compared to those without differed in measures of mood and functioning including the functional impact of fibromyalgia (FIQ‐R), work and social functioning (WSAS), and depression (PHQ‐9). Given previous evidence on the difference between people with multiple DGBIs and those without (Sperber et al. 2022), participants with more than one DGBI were also compared with participants without any DGBIs.

Pearson's correlations were calculated to examine the correlations among GI symptom severity (GSRS) and fibromyalgia symptom severity (the symptom severity subscale of FIQ‐R), PF (Psy‐Flex), and measures of mood and daily functioning.

Cross‐sectional mediation analyses were conducted to explore whether PF (Psy‐Flex) buffered the associations between GI symptom severity (GSRS) and measures of mood and daily functioning, using the PROCESS extension in SPSS version 4.2. Indirect effects were estimated using bias‐corrected bootstrap confidence intervals based on 5000 bootstrap samples. Background variables, including age, education, working status, pain duration, and pain intensity that were significantly associated with the outcome variables, were included in the mediation models as covariates. Missing data were deleted pairwise.

Continuous study variables that were included in the main analyses were examined for missing data and outliers. Histogram and Q‐Q plot were examined alongside skewness and kurtosis values for normality. FIQ had no missing data; GSRS was missing data for 6 participants, WSAS, PHQ‐9 and PF were missing data for 10, 12 and 21 participants, respectively. One outlier was identified for FIQ and five for WSAS, which were removed. All variables showed approximately symmetric distribution, with skewness and kurtosis values within commonly accepted thresholds, and Q–Q plots indicating only minor deviations from the diagonal line.

## 3 Results

### 3.1 The Rate of DGBI in the Sample

Participants reported having received diagnoses for one to twelve diagnosed DGBIs. Of all participants, 79.2% reported a diagnosis of at least one DGBI, and 43% reported more than one DGBI. Among participants with more than one DGBI, 78 had two DGBIs, 46 three, 21 four, 12 five, 7 six, 3 seven, 1 ten, 1 eleven, and 1 twelve. IBS was the most prevalent DGBI, diagnosed in 70.38% of the participants, followed by Reflux Hypersensitivity, Functional Heartburn, Functional Constipation, and Functional Abdominal Bloating, each reported by approximately 10%–19% of the participants. Table 2 shows the number of participants and the rate for each DGBI.

**TABLE 2:** The number of participants and rate for each disorder of gut–brain interaction.

Disorder of gut–brain interaction	*n*	%
Irritable Bowel Syndrome	278	70.38%
Reflux hypersensitivity	75	18.99%
Functional heartburn	60	15.19%
Functional constipation	44	11.14%
Functional abdominal bloating/distension	40	10.13%
Functional diarrhoea	25	6.33%
Opioid‐induced constipation	24	6.08%
Biliary pain (functional gallbladder disorder, functional biliary sphincter of oddi disorder)	23	5.82%
Faecal incontinence	22	5.57%
Functional chest pain	17	4.30%
Belching disorders (excessive supragastric belching, excessive gastric belching)	14	3.54%
Unspecified functional bowel disorder	13	3.29%
Functional dysphagia	9	2.28%
Functional dyspepsia (postprandial distress syndrome, epigastric pain syndrome)	8	2.03%
Nausea and vomiting disorder (chronic nausea vomiting syndrome, cyclic vomiting syndrome, cannabinoid hyperemesis syndrome)	7	1.77%
Functional defecation disorders (inadequate defecatory propulsion, dyssynergic defecation)	5	1.27%
Globus	4	1.01%
Functional anorectal pain (levator ani syndrome, unspecified functional anorectal pain, proctalgia fugax)	4	1.01%
Centrally mediated abdominal pain syndrome	3	0.76%
Narcotic bowel syndrome/Opioid‐induced GI hyperalgesia	2	0.51%

### 3.2 Comparison of Participants With and Without Self‐Reported DGBI Diagnose

Participants with at least one DGBI and those without any DGBIs did not significantly differ in the functional impact of fibromyalgia, *t* (393) = −1.56, *p* = 0.12, work and social functioning, *t* (383) = −1.63, *p* = 0.10, or depression, *t* (381) = −1.09, *p* = 0.28. However, participants with more than one DGBI showed significantly greater functional impact of fibromyalgia, *t* (250) = −2.67, *p* < 0.05, and more impaired work and social functioning, *t* (244) = −2.57, *p* < 0.05, compared with those without any DGBIs. Table 3 shows the *t*‐test results.

**TABLE 3:** *T*‐tests results for the comparisons between participants with DGBIs and those without any.

	At least one DGBI vs. no DGBI					More than one DGBI vs. no DGBI				
	Mean difference	*t*	df	*d*	*p*	Mean difference	*t*	df	*d*	*p*
FIQ‐R	−3.43	−1.56	393	−0.19	0.12	−6.06	−2.67	250	−0.36	0.01
WSAS	−1.61	−1.63	383	−0.21	0.10	−2.65	−2.57	244	−0.35	0.01
PHQ‐9	−0.82	−1.09	381	−0.14	0.28	−1.33	−1.64	243	−0.22	0.10
Psy‐Flex	−5.24	−0.28	102.91	−0.04	0.78	−1.28	−0.07	236	−0.01	0.95

Abbreviations: FIQ‐R, Fibromyalgia Impact Questionnaire‐Revised; PHQ‐9, Patient Health Questionnaire‐9; WASA, Work and Social Adjustment Scale.

### 3.3 The Correlations Between GI Symptom Severity, Fibromyalgia Symptom Severity, PF and Measures of Mood and Daily Functioning

Table 4 shows the correlations between GI symptom severity, fibromyalgia symptom severity, PF and measures of mood and daily functioning. GI symptom severity was significantly and positively correlated with fibromyalgia symptom severity, *r* = 0.59, *p* < 0.001. More severe GI symptoms were statistically significantly correlated with lower PF, r = −0.22, *p* < 0.001, and higher functional impact of fibromyalgia, more impaired work and social functioning and more severe depression, *r* = 0.41 to 0.56, *p* < 0.001. Greater PF was significantly correlated with less functional impact of fibromyalgia, better work and social functioning and less severe depression, *r* = 0.28 to 0.42, *p* < 0.001.

**TABLE 4:** The correlations between GI symptom severity, fibromyalgia symptom severity, psychological flexibility and measures of functioning.

	FM Symptom severity	Functional impact of FM	Work and social functioning	Depression	Psychological flexibility
GI symptom severity	0.59	0.56	0.41	0.54	−0.22
FM symptom severity			0.67	0.71	−0.33
Psychological flexibility	−0.33	−0.31	−0.28	−0.41	

*Note:* All *p* < 0.001, *N* = 369–395. The correlation between fibromyalgia symptom severity and functional impact of fibromyalgia was not examined, as the fibromyalgia symptom severity scores were derived from the measure of the functional impact of fibromyalgia (FIQ‐R).

### 3.4 The Buffering Role of PF in the Relationship Between GI Symptom Severity and Measures of Mood and Daily Functioning

The associations between background variables, including age, education, working status, pain duration and pain intensity, and outcome variables were first examined to identify potential covariates. Gender and ethnicity were not examined as the vast majority of participants were women (97%) and white (96.5%). Work status was re‐categorised into working or studying and not working or studying to facilitate analysis.

Age and education were not correlated with any outcome variables, and participants with different working status differ in all outcome variables, with participants who were working or studying showing lower functional impact of fibromyalgia, *t* (375.06) = 5.26, *p* < 0.001, less impaired work and social functioning, *t* (341.7) = 7.23, *p* < 0.001, and less severe depression, *t* (381) = 2.8, *p* < 0.01, Pain intensity was significantly correlated with FIQ scores, *r* = 0.65, *p* < 0.001, PHQ‐9 scores, *r* = 0.46, *p* < 0.001, and WSAS scores, *r* = 0.51, *p* < 0.001. Pain duration was significantly correlated with FIQ scores, *r* = 0.16, *p* < 0.001, and WSAS scores, *r* = 0.17, *p* < 0.001. Therefore, working status and pain intensity were included as co‐variates in all mediation models. Albeit the weak correlations between pain duration and FIQ scores and WSAS scores, pain duration was included as a co‐variate in the mediation models with FIQ‐R scores and WSAS scores as dependent variables.

**FIGURE 1:**
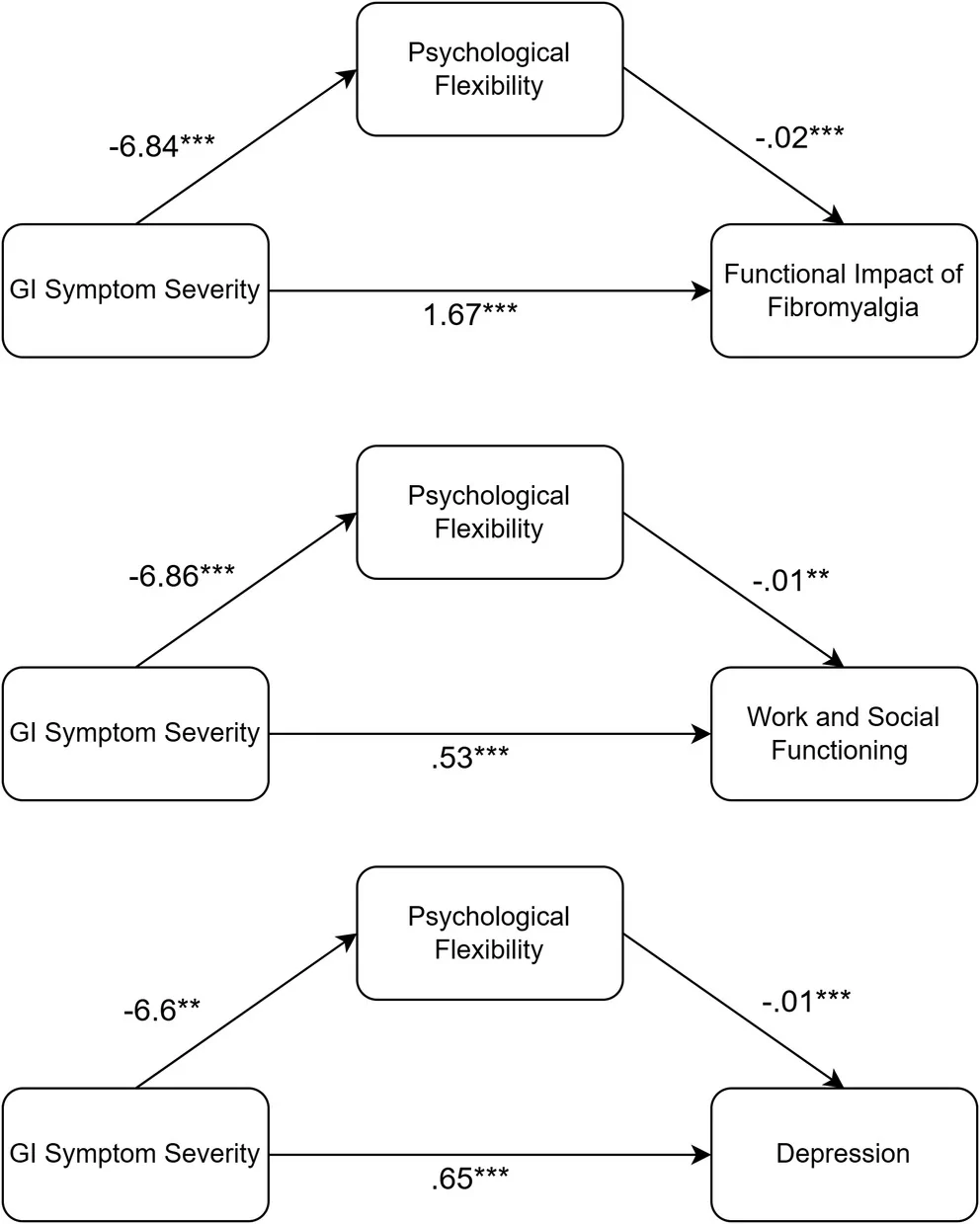
Mediation models illustrating the buffering effects of psychological flexibility on the relationship between gastrointestinal (GI) symptom severity and measures of mood and daily functioning.

Figure 1 illustrates the mediation models showing the regression coefficients for the direct effect of GI symptom severity on PF (a), direct effect of PF on measures of mood and daily functioning controlling for GI symptom severity (*b*), and the direct effect of GI symptoms severity on measures of mood and daily functioning controlling for PF (c′). Although not part of the study objectives, the results of mediation analyses with fibromyalgia symptom severity as the independent variable are provided in the Supporting Information for readers interested in further details.

The total effect of GI symptom severity on the functional impact of fibromyalgia was significant, *b* = 1.8, *p* < 0.001. The indirect effect of GI symptom severity on the functional impact of fibromyalgia through PF was significant, *b* = 0.13, CI [0.0378, 0.2794], and the direct effect of GI symptom severity on the functional impact of fibromyalgia was significant after controlling for PF, *b* = 1.67, *p* < 0.001. Therefore, PF partially statistically mediated the effect of GI symptom severity on the functional impact of fibromyalgia, and 7.2% of the total effect was explained by the indirect effect.

The total effect of GI symptom severity on work and social functioning was significant, *b* = 0.59, *p* < 0.001. The indirect effect of GI symptom severity on work and social functioning through PF was significant, *b* = 0.05, CI [0.0122, 0.1158], and the direct effect of GI symptom severity on work and social functioning was significant after controlling for PF, *b* = 0.53, *p* < 0.001. Therefore, PF partially statistically mediated the effect of GI symptom severity on work and social functioning, and 8.6% of the total effect was explained by the indirect effect.

The total effect of GI symptom severity on depression was significant, *b* = 0.73, *p* < 0.001. The indirect effect of GI symptom severity on depression through PF was significant, *b* = 0.08, CI [0.0263, 0.1446], and the direct effect of GI symptom severity on depression was significant after controlling for PF, *b* = 0.65, *p* < 0.001. Therefore, PF partially statistically mediated the effect of GI symptom severity on depression, and 11% of the total effect was explained by the indirect effect.

## 4 Discussion

The aim of this study was to investigate the occurrence of DGBIs in fibromyalgia, potential consequences for people when this happens, and a potential protective factor, psychological flexibility. In this sample, nearly 80% of the individuals with fibromyalgia had at least one DGBI (objective 1). Participants with more than one DGBI showed significantly greater functional impact of fibromyalgia and more impaired work and social functioning compared with those without any DGBIs. More severe GI symptoms were associated with more severe fibromyalgia symptoms, worse mood and daily functioning, and lower PF. Moreover, greater PF was associated with better mood and daily functioning (objective 2). Last but not least, PF buffered the relationship between GI symptom severity and all measures of mood and daily functioning (objective 3).

To our knowledge, this is the first study investigating the role of DGBIs in relation to PF and functioning in people with fibromyalgia. As expected, PF was found to be associated with all measures of mood and daily functioning in this study, supporting findings from previous research. In a study of ACT for IBS (Ferreira et al. 2018), IBS acceptance, one of the PF processes, was found to predict all measures of treatment outcomes, including quality of life, anxiety, and avoidant behaviours. Cross‐sectional studies have also demonstrated the associations of PF with emotional, physical or daily functioning in people with chronic abdominal pain (Yu et al. 2021), and people with fibromyalgia (Rodero et al. 2011; Yu et al. 2017). One randomised controlled trial (RCT) also found the association between the change in PF and the changes in pain, pain‐related disability, and depression (Wicksell et al. 2013; Yu et al. 2017). These findings suggest a potential role of PF in mitigating the impact of comorbid DGBI and fibromyalgia.

It is worth noting that PF accounted for small to moderate proportions of variance in mood and daily functioning, suggesting other factors such as symptoms may also play a role in functioning in this population. In this study, the direct effect of GI symptom severity and fibromyalgia symptom severity on all measures of mood and daily functioning controlling for PF was significant, supporting the role of symptoms in relation to functioning. Diet has also been identified to influence gut functioning in people with DGBIs (So and Tuck 2024). Contextual factors such as healthcare may also explain variance in functioning. For example, a large national survey study identified significant problems in the UK healthcare services provision and access for people with fibromyalgia (Wilson et al. 2022), which can exert a negative impact on functioning in this under‐served population.

Both fibromyalgia and DGBIs involve central sensitization, interoceptive dysregulation, and avoidance‐oriented coping. The PF model, underpinning ACT, is theoretically and clinically positioned to influence these processes by reducing attentional hypervigilance, affective amplification, and behaviour restriction. Indeed, in our cross‐sectional mediation analyses, after controlling for pain, pain duration and/or working status, PF still partially statistically mediated the relationships between GI symptom severity and all measures of functioning, suggesting a potential buffering role of PF in the relationship between GI symptoms and mood and daily functioning and the potential utility of ACT in this population.

In fact, ACT has been applied in the management of fibromyalgia (Eastwood and Godfrey 2024) and some GI conditions including IBS and IBD (Marchese et al. 2024), leading to improvements in a range of outcomes, including pain, fatigue, disability, anxiety, depression, overall functioning, GI symptoms and quality of life. Notably, rather than seeking to reduce or eliminate symptoms, ACT targets common underlying processes across diagnoses—psychological flexibility—through which symptoms influence health and wellbeing, thereby improving clinical outcomes. The ‘trans‐diagnostic’ nature of ACT may mean it is particularly suitable for addressing complex comorbid problems. Further studies examining the application of ACT to fibromyalgia comorbid with DGBIs are urgently needed to address the challenges this population faces.

While PF was significantly associated with symptom severity, the correlation between PF and GI symptom severity was also relatively small. This pattern is theoretically consistent, as PF is the underpinning process through which symptoms exert influence on functioning, but not a process primarily associated with symptoms per se. As such, stronger associations are typically observed with functioning than with symptom severity.

This is also the first study examining the prevalence of DGBIs in fibromyalgia in a large community sample. While the prevalence of DGBIs in people with fibromyalgia has only been estimated in a few small studies, the high rate of DGBIs in individuals with fibromyalgia observed in this study appears to be consistent with previous estimates. It was found in one study (*N* = 200) that 98% of the fibromyalgia patients had at least one DGBI, while this rate was only 39% in controls (Almansa et al. 2009). In another study of 111 individuals with fibromyalgia, 93% had at least one DGBI (Erdrich and Harnett 2025). Despite the high prevalence, research on DGBIs comorbid with fibromyalgia has been relatively limited and largely focused on IBS. While IBS was the most common DGBI in this study, other DGBIs such as reflux hypersensitivity (18.99%) and functional heartburn (15.19%) were also frequently present, and nearly half of the participants had more than one DGBI. Research that comprehensively investigates DGBIs in people with fibromyalgia is needed to understand the scale and impact of the problem.

Unsurprisingly, participants with more than one comorbid DGBI reported greater functional impact of fibromyalgia, and worse work and social functioning, compared with those without any DGBIs. A recent Rome Foundation report has identified the association of a higher number of DGBI diagnoses with increased symptom severity, healthcare use and comorbid psychological problems, and decreased quality of life (Barbara et al. 2025).

However, participants with only one DGBI did not differ from participants who did not have any DGBIs in any measures of mood and daily functioning. To further explore the unexpected finding, we examined participants with only one DGBI. Out of 143 participants with only one DGBI, 123 reported having IBS, while few or no participants reported having any of the other disorders. It is plausible that the challenges of comorbid IBS alone do not exert substantially greater influence on functioning among individuals with fibromyalgia, suggesting the need to comprehensively examine DGBIs in people with fibromyalgia. It is also possible that some participants who reported no DGBIs may nevertheless have experienced symptoms consistent with these conditions that had not been formally diagnosed. Nevertheless, our finding appears to be consistent with the finding from a global survey of DGBIs including over 54,000 adults, showing that overlapping DGBIs were associated with increased health care utilisation, comorbid psychological problems and IBS severity (Sperber et al. 2022).

Consistently, GI symptom severity was statistically significantly associated with all measures of mood and daily functioning, with moderate to large effect. This finding resonates with a pilot study of an ACT‐based treatment for IBS (Ferreira et al. 2018) where symptom severity was found to predict quality of life, anxiety, and avoidance behaviours. Results from the Rome Foundation Global Study also suggested that people with DGBIs are more likely to have lower health‐related quality of life and more doctor visits (Sperber et al. 2021).

In line with our finding that fibromyalgia symptoms severity was significantly associated with mood and daily functioning with large effects, evidence has also shown associations between fibromyalgia symptoms such as pain and fatigue with indicators of functioning and wellbeing (e.g., (Alvarez et al. 2022; Bartley et al. 2018; Humphrey et al. 2010; Singh et al. 2024)). Given the considerable impact of either condition, the experience of DGBIs and fibromyalgia in combination seems to exacerbate the suffering of affected individuals.

This study has limitations. First and foremost, the cross‐sectional design of the study limited our ability to infer any causal relations, including the ‘mediating’ role of PF in the relationships between GI symptom severity and measures of mood and daily functioning. Longitudinal studies with experimental design are needed to understand the role of PF, which in turn can inform treatment development for people with co‐occurring fibromyalgia and DGBIs.

Second, the sample was recruited from relevant online support groups and organisations, and it is unclear whether individuals seeking support online are representative of the larger population, which limited the generalizability of the findings. For instance, individuals who seek such support may be more severely affected, hence representing individuals with high symptom burden. Further studies in different contexts are needed to facilitate more comprehensive and fine‐grained understanding of the problem. Relatedly, due to the online recruitment approach, participants' diagnoses could not be verified. However, the diagnosis of fibromyalgia remains inherently challenging because of its complexity. Stigma and other obstacles can make it even harder for individuals to seek or obtain an official diagnosis (Bidari et al. 2018). Nevertheless, 98.5% of the participants reported that their fibromyalgia was diagnosed by a clinician.

The self‐reported diagnosis of DGBI rather than assessment via the Rome IV diagnostic questionnaires is another limitation. This is particularly relevant for non‐IBS DGBIs, which are less commonly discussed explicitly in clinical encounters and may not be consistently coded in medical records. As such, some of the self‐reported DGBI diagnoses may reflect perceived or recalled diagnoses rather than strictly defined Rome IV DGBIs. Although self‐reported diagnoses are not equivalent to Rome IV diagnoses, it has been shown that self‐reported IBS identify individuals with substantial gastrointestinal symptom burden, impaired quality of life and increased healthcare utilisation, despite only partial overlap with Rome IV‐defined IBS (Brunkwall et al. 2021). Nevertheless, future studies incorporating established diagnostic criteria in the screening process could enhance methodological rigour and strengthen the validity of findings.

Next, while fibromyalgia is commonly more prevalent among women, the dominantly white female sample limited the generalisability of the findings. More diverse recruitment pathways need to be used in future studies to enhance inclusion and explore the general applicability of findings. Finally, while DGBIs commonly co‐occur with organic GI disorders, information on organic GI disorders was not collected due to the focus on DGBIs and the exploratory nature of this preliminary study. Further studies that comprehensively collect information on related health problems can allow more fine‐grained analyses and deepen our understanding of complex comorbidities in people with fibromyalgia, which in turn can inform targeted treatment development.

## 5 Conclusions

Most people with fibromyalgia have co‐occurring DGBIs, and these are associated with greater impact on key aspects of functioning for people with fibromyalgia. PF may buffer the impact of symptom severity and functioning, although the magnitude of relations observed here was modest. Hence ACT, based on PF, may provide benefits for people facing the challenges of co‐occurring fibromyalgia and DGBIs. Longitudinal studies with experimental designs are needed to further investigate the mediating role of PF and other protective factors to inform treatment development. Psychological treatments with a focus on co‐occurrence and interactions between fibromyalgia and DGBIs are needed to address the challenges faced by this population.

## Author Contributions

All authors discussed the results and commented on the manuscript. All authors substantially contributed to the conception and design of the project. All authors substantially contributed to critically revising the draft and the final approval of the revision to be published. In addition, L.Y. significantly contributed to drafting the article and analysis and interpretation of the data, and K.K. significantly contributed to data collection.

## Funding

The authors have nothing to report.

## Conflicts of Interest

The authors declare no conflicts of interest.

## Supporting information


**Table S1:** Mediation coefficients.
