# Supplementary material for: Preliminary Investigation of Disorders of Gut–Brain Interaction and Their Associations With Psychological Flexibility and Daily Functioning in People With Fibromyalgia
Source: Eur J Pain. 2026 Sep 3;30(8):e70378. doi: 10.1002/ejp.70378 (PMC13540553; doi:10.1002/ejp.70378)
Supplement: Supplementary file 1 [file EJP-30-0-s001.docx]

Supplementary: The results of mediation analyses examining the buffering role of PF in the relationship between fibromyalgia symptom severity and measures of mood and daily functioning

The functional impact of fibromyalgia measured by the fibromyalgia impact questionnaire (FIQ) was not examined in the mediation analyses, as fibromyalgia symptom severity scores were derived from the FIQ. Working status and pain intensity was included as a co-variate in both mediation models, and pain duration was also included as a co-variate in the mediation model with WSAS as dependent variable.

Table S1 Mediation coefficients

|  | Path a  (direct effect of fibromyalgia symptom severity on PF) | | Path b  (direct effect of PF on functioning) | | Path c’  (direct effect of fibromyalgia symptoms severity on functioning) | | a*b  (indirect effect of fibromyalgia symptom severity on functioning) | |
| --- | --- | --- | --- | --- | --- | --- | --- | --- |
|  |  |  |  |  |  |  |  |  |
|  | b | p | b | p | b | p | b | Bootstrapped CI |
| PHQ-9 | -2.80 | <.001 | -.01 | <.001 | .22 | <.001 | .03 | .0128, .0389 |
| WSAS | -2.87 | <.001 | -.003 | .17 | .26 | <.001 | .01 | -.0036, .0252 |

Note. PHQ-9: Patient Health Questionnaire-9; WASA: Work and Social Adjustment Scale. CI: Confidence Intervals. 5,000 bootstrap resamples were used.

The total effect of fibromyalgia symptom severity on depression was significant, b=.25, p<.001. The indirect effect of fibromyalgia symptom severity on depression through PF was significant, b=.03, and the direct effect of fibromyalgia symptom severity on depression was significant after controlling for PF, b=.22, p<.001, suggesting PF partially mediated the effect of fibromyalgia symptom severity on depression.

The total effect of fibromyalgia symptom severity on work and social functioning was significant, b=.27, p<.001. The direct effect of fibromyalgia symptom severity on work and social functioning was significant after controlling for PF, b=.26, p<.001. However, the indirect effect of fibromyalgia symptom severity on work and social functioning through PF was not significant, b=.01., suggesting PF did not mediated the effect of fibromyalgia symptom severity on work and social functioning.
